# CONCUR: quick and robust calculation of codon usage from ribosome profiling data

**DOI:** 10.1093/bioinformatics/btaa733

**Published:** 2020-08-31

**Authors:** Michaela Frye, Susanne Bornelöv

**Affiliations:** Cell Biology and Tumor Biology, German Cancer Research Center (DKFZ), 69120 Heidelberg, Germany; Wellcome – MRC Cambridge Stem Cell Institute, University of Cambridge, Cambridge CB2 0AW, UK

## Abstract

**Summary:**

CONCUR is a standalone tool for codon usage analysis in ribosome profiling experiments. CONCUR uses the aligned reads in BAM format to estimate codon counts at the ribosome E-, P- and A-sites and at flanking positions.

**Availability and implementation:**

CONCUR is written in Perl and is freely available at https://github.com/susbo/concur.

**Supplementary information:**

Supplementary data are available at *Bioinformatics* online.

## 1 Introduction

Ribosome profiling (Ribo-seq) relies on mRNA sequencing to measure protein translation. Because mRNAs located within a ribosome are protected from nuclease digestion, they can be captured and sequenced to reveal a snapshot of active translation (Ingolia *et al.*, 2009). Ribo-seq can be used to study protein synthesis, translational dynamics and decoding. During translation elongation, the ribosome moves three nucleotides (nts) at a time when shifting from one codon to the next, thereby giving a characteristic 3-nt periodicity to the data. This periodicity enables the computational mapping of ribosomes at single-codon resolution.

Importantly, the more time the ribosome spends at a given codon, the more likely that codon it is to be captured and sequenced. By calculating and comparing codon counts across different samples, differences in codon–anticodon pairing and amino acid incorporation can be identified.

However, challenges arise from batch effects due to different experimental approaches, resulting in differently sized ribosome-protected mRNA fragments. Finding the correct reading frame and estimating codon frequencies is currently a time-consuming task and many steps have to be resolved manually. To streamline this process, we have developed CONCUR, a standalone tool for fast and efficient codon usage estimation. The only required input is a BAM file with aligned reads. CONCUR currently supports human, mouse, yeast and rat data but additional genomes can easily be installed.

## 2 Materials and methods

Although most Ribo-seq data show 3-nt periodicity, the reading frame differs between experiments and read lengths. CONCUR calculates the reading frame periodicity for each read length and estimates the shift required for the ribosome P-site to be located at the same position in each read. The read lengths with the strongest periodicity are selected and shifted before calculating the overall codon usage.

### 2.1 Initial read shift estimation

Reads, which are 20–50nts in length, are included in the analysis, with the typical read length being around 28nts. Aligned reads that intersect annotated exons are identified and their 5′ reading frame is calculated. Reads mapping to the mitochondria are excluded. Reads mapping within 100nts of a start codon are extracted and used to identify the relative shift of each read length and frame. During translation, the ribosome stalls with the start codon positioned in its P-site. Protecting approximately 12nts upstream of its P-site from RNase digestion, this creates a distinctive peak upstream of the translation initiation site (TIS). However, the location of the peak differs slightly for each read length and must therefore be estimated from the data.

To find the optimal offset, we count the number of 5′ ends mapping to each position around the expected peak 12nts upstream of the TIS. Each read length and frame are treated separately and the best shift, *i*, is estimated as

$$\underset{-6<i<6}{\mathrm{argmax}}\{2c_{i}-\frac{c_{i-9}}{9}-\frac{c_{i-6}}{3}-c_{i-3}-\frac{c_{i+3}}{3}-\frac{c_{i+6}}{9}-\frac{c_{i+9}}{9}\},$$

where *c_i_* is the number of 5′ ends mapping to position -12+*i*. Only positions defining a local counts maxima, $c_{i-3}<c_{i}>c_{i+3}$, are considered.

To avoid inconclusive data, only read lengths and shifts where $c_{i}/(c_{i-3}+c_{i}+c_{i+3})>0.5$ and with at least 1000 reads near the TIS are used in the following analysis. Those constrains are necessary to exclude read lengths causing noise. Lengths around 27–33nts are typically selected in this step and multiple frames are often informative.

### 2.2 Validation and final selection of read shifts

The selected read lengths and frames are used to calculate codon usage at the E-, P- and A-sites as well as three flanking positions in each direction. If all shifts were successfully predicted, the calculated codon frequencies at the P- and A-site should—regardless of their read length and frame—only be similar to frequencies at the same site. In addition, all other sites should show codon frequencies similar to each other and to the transcriptome-wide codon distribution. A similar strategy to select the offsets is used by RUST (O’Connor *et al.*, 2016).

#### 2.2.1 Correlation to transcriptome-wise counts

To confirm that the predicted codon counts at the P- and A-sites both differ from the transcriptome-wide distribution, we defined a *read set*, *R_lf_*, as all reads of a specific length, l∈{20,…,50}, and frame, f∈{0,1,2}. Codon counts for read set *R_lf_* at site s∈{−3,−2,−1,E,P,A,+1,+2,+3} are represented as $C_{lf}^{s}$. The read sets were scored based on how they correlated to the transcriptome-wide codon distribution. The mean correlation at the P- and A-sites is compared to the -2, -1, E, +1 and +2 sites:

$$S_{c}(C_{lf})=\sum_{i\in \{-2,-1,E,+1,+2\}}\frac{\rho (C_{lf}^{i},C_{\text{bg}})}{5}-\sum_{i\in \{P,A\}}\frac{\rho (C_{lf}^{i},C_{\text{bg}})}{2}$$

where $\rho (C,C_{\text{bg}})$ is the correlation between the codon counts *C* and the transcriptome-wide background distribution, $C_{\text{bg}}$.

If the P- and A-sites had higher mean correlation than the flanking positions, i.e. if $S_{c}<0$, the read set was discarded. The read set with the highest score was identified as the best read set, and $C_{\text{best}}^{P}$ and $C_{\text{best}}^{A}$ refer to the codon counts for that read set at positions P and A, respectively.

#### 2.2.2 Correlation to the best read sets

Next, each read set was evaluated based on how well it agreed with the best read sets at the P- and A-sites. A read set was kept only if $C_{\text{best}}^{P}$ had the highest correlation to the P-site in the tested read set, and similarly, if $C_{\text{best}}^{A}$ has the highest correlation to the A-site of the tested read set:

$$\{\begin{array}{c} \overset{}{\underset{i\in \{-3,-2,-1,E,P,A,+1,+2,+3\}}{\mathrm{argmax}}}\rho (C_{lf}^{i},C_{\text{best}}^{P})=P\\ \overset{}{\underset{i\in \{-3,-2,-1,E,P,A,+1,+2,+3\}}{\mathrm{argmax}}}\rho (C_{lf}^{i},C_{\text{best}}^{A})=A \end{array}$$

#### 2.2.3 Removal of outliers

Finally, to verify that none of the selected read sets have very different codon counts at the P- or A-site, we defined a rank-based score, $S_{r}(C_{lf}^{s})$, for each site *s* as:

$$S_{r}(C_{lf}^{s})=\sum_{C_{l\prime f\prime }^{s}}\{\begin{array}{cc} 1 & \text{if}\;\text{rank}(C_{l\prime f\prime }^{s})\leq n\\ 0 & \text{otherwise}\; \end{array}$$

where $\text{rank}(C_{l\prime f\prime }^{s})$ is the rank of $\rho (C_{lf}^{s},C_{l\prime f\prime }^{s})$ among all possible $\rho (C_{lf}^{s},C)$—with the highest correlation giving the lowest rank (1)—and *n* is the number of read sets from site *s*. Intuitively, this score reflects how many of the most correlated sets of codon counts that derive from the expected site. A read set *R_lf_* is kept if its scores at the P- and A-sites are at least half that of the highest score, i.e. if $S_{r}(C_{lf}^{s})\geq \frac{1}{2}S_{r}^{\text{max}}(C_{l\prime f\prime }^{s})$ for s∈{P,A}.

Finally, codon usage was calculated using only reads from read sets that passed all three filters.

## 3 Results

### 3.1 Usage examples on real data

CONCUR was used to replicate the results of two published studies. The first study by Nedialkova *et al.* (2015) examined yeast in which a tRNA anticodon modification was disrupted, resulting in slower translation at cognate codons *in vivo*. The second study by Bornelöv *et al.* (2019) characterized codon usage changes during differentiation of human pluripotent stem cells. Ribo-seq data were downloaded and pre-processed and CONCUR was run on the resulting BAM files. For the tested datasets, CONCUR reproduced the published results or was able to detect a stronger effect. SeeFigure 1 for an example. More details and results are available in Supplementary Section S1 in Supplementary Materials, including some run-time examples.

**Fig. 1.**
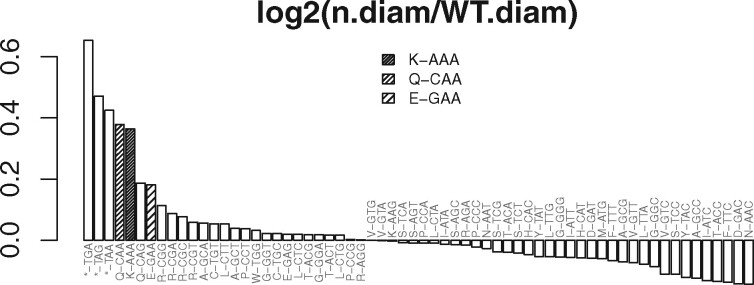
Codon enrichments in yeast lacking the *ncs2* gene compared to wild-type controls at the ribosome A-site during stress (30-min diamide treatment). The three codons that require a modified tRNA are highlighted. The stop codon enrichment suggests an additional effect on translational termination. Values shown are log2-transformed

### 3.2 Differences to existing tools

To our knowledge, CONCUR is the first tool dedicated to codon usage estimation. However, some existing tools and pipelines are capable of P-site offset calculation and/or codon usage estimation. An overview of existing tools and key features and differences are provided in Supplementary Sections S2.1 and S2.2 in Supplementary Materials.

In contrast to the other tools, CONCUR can handle multiple frames for the same read length. This approach has previously only been used by Ribodeblur, an offset estimation tool developed specifically for yeast (Wang *et al.*, 2017). Inclusion of multiple frames and the extensive filters and validation steps in CONCUR provided more consistent results on the test data compared with several existing tools. This is discussed further in Supplementary Sections S2.2 and S2.3 in Supplementary Materials.

## 4 Conclusion

We present CONCUR, a tool aimed to simplify and standardize codon usage analysis. CONCUR has been extensively tested on in-house and publicly available datasets. The only required input is a BAM file with alignments to a reference genome, making the process very quick and robust.

## Funding

This work was supported by Cancer Research UK [C10701/A15181] and a core support grant from the Wellcome Trust and Medical Research Council to the WT-MRC Cambridge Stem Cell Institute.

*Conflict of Interest*: none declared.

## Supplementary Material

btaa733_Supplementary_Data
