# Supplementary material for: CONCUR: quick and robust calculation of codon usage from ribosome profiling data
Source: Bioinformatics. 2020 Aug 31;37(5):717–9. doi: 10.1093/bioinformatics/btaa733 (PMC8097682; doi:10.1093/bioinformatics/btaa733)
Supplement: btaa733_Supplementary_Data [file btaa733_supplementary_data.pdf]

Supplementary materials for *"CONCUR: quick and robust calculation of codon usage from ribosome profiling data"*

Michaela Frye[1] Susanne Bornelöv[2,*]

[1]German Cancer Research Center (DKFZ), Im Neuenheimer Feld 280, 69120, Heidelberg, Germany.
[2]Wellcome - MRC Cambridge Stem Cell Institute, University of Cambridge, Puddicombe Way, Cambridge, CB2 0AW, UK.

*To whom correspondence should be addressed.

Contents

1 Running CONCUR on published data 2
1.1 Codon usage changes in differentiating human cells 2
1.1.1 Data and processing 2
1.1.2 Results 2
1.2 Disruption of tRNA thiolation in yeast 6
1.2.1 Data and processing 6
1.2.2 Results 6
1.2.3 Runtime 11

2 Comparing CONCUR to other methods 11
2.1 Overview of methods for P-site offset estimation 11
2.2 Key features and differences to other tools 12
2.2.1 Simple processing and input format 12
2.2.2 Focus on codon usage estimates 12
2.2.3 Correct inclusion of multiple frames per read length . . . 13
2.3 Benchmarking of P-site offset estimation 13
2.3.1 Running CONCUR, Plastid, ORFik, and Shoelaces 13
2.3.2 Benchmarking results 14

List of Figures

S1 Correlation between selected read sets in human 3
S2 Codon counts across all positions in human 4
S3 Codon enrichment differences during differentiation 5

S4 Correlation between selected read sets in yeast 7
S5 Codon counts across all positions in yeast 8
S6 Codon enrichment differences following *ncs2* deletion 9
S7 Codon enrichment differences following elp6 deletion 10
S8 Runtime 11

List of Tables

S1 Selection of reads in human 3
S2 Selection of reads in yeast 6
S3 Tool and pipelines that perform P-site offset identification 12
S4 P-site offset estimation in three *ncs2* knockout samples 15
S5 P-site offset estimation in three wildtype samples 16
S6 Benchmarking results 17

1 Running CONCUR on published data

1.1 Codon usage changes in differentiating human cells

1.1.1 Data and processing

Self-renewing human stem cells show a specific codon usage profile, which changes during differentiation (Bornelöv *et al.*, 2019). Specifically, codons that rely on the adenine to inosine modification at position 34 (A34I)–the wobble position–in tRNA anticodon loops, are more efficiently translated in the self-renewing state. This was previously measured as a reduction of the A34I modification levels in the differentiated state and an enrichment of ribosome profiling reads corresponding to those codons at the ribosome A-site (Bornelöv *et al.*, 2019).

To confirm that CONCUR detected those enriched codons, we used the published ribosome profiling data (data accessible at NCBI GEO database, accession GSE123611). Trim_galore! [`https://www.bioinformatics.babraham.ac.uk/projects/trim_galore`] was used to trim small RNA adapters and trimmed reads shorter than 20 nt were discarded. Alignment was done using TopHat2 (v2.1.0). First, the reads were aligned to a set of known rRNA and tRNA genes (selected from the UCSC RepeatMasker tracks), this was followed by alignment of all unmapped reads to the human reference genome (hg38). An index with known transcripts based on Gencode v23 annotations were used for the genome alignment, novel splice junctions were permitted, and multi-mapping reads were discarded. The resulting BAM files were used as input to CONCUR.

1.1.2 Results

CONCUR was used to select read lengths and frames (Table S1), and to calculate the best shifts. The codon counts derived from the selected reads displayed a strong correlation with each other at both the P- and the A-sites (Fig. S1).

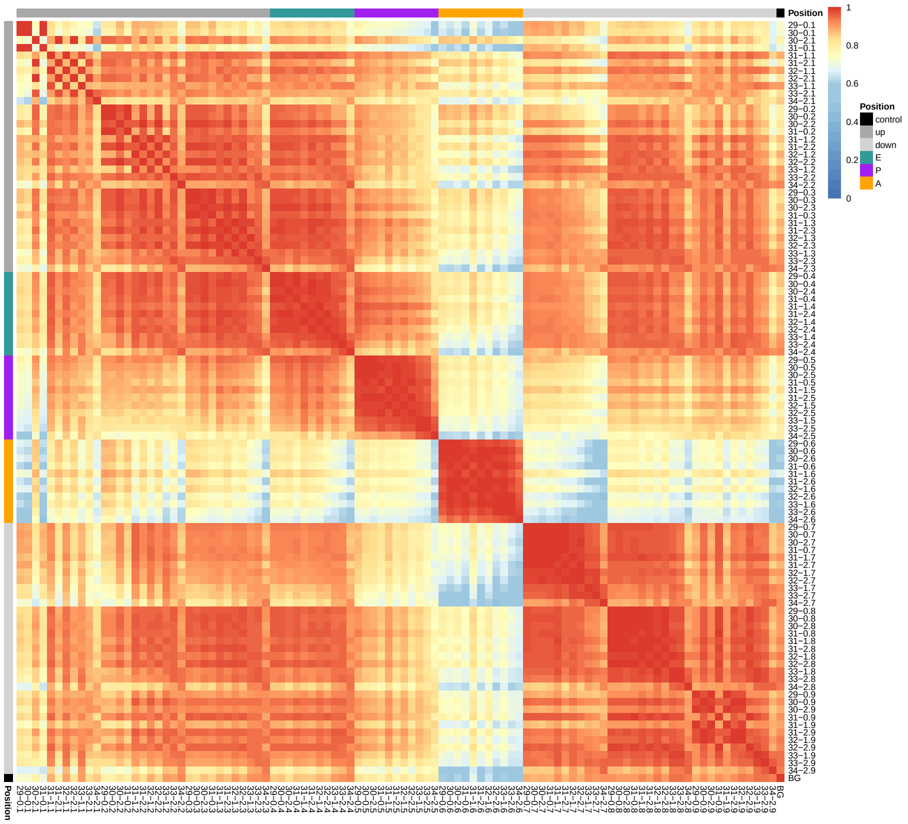

Figure S1: Correlation between selected read sets for the *Differentiating 1* sample across all read positions.

An example of the resulting codon counts across all positions is shown in Fig. S2. These two types of figures (Fig. S1 and Fig. S2) suitable as a quality control are automatically created by CONCUR unless the ”--noR” option is used.

Table S1: Selection of reads in differentiation experiment

**Sample**	**Selected lengths and frames**
Self-renewing 1	27-0 28-0 29-0 29-2 30-0 30-2 31-0 31-1 31-2 32-0 32-1 32-2 33-1 33-2 34-1 34-2
Self-renewing 2	27-0 28-0 29-0 29-2 30-0 30-2 31-1 31-2 32-1 32-2 33-1 33-2
Self-renewing 3	27-0 28-0 29-0 29-2 30-0 30-2 31-1 31-2 32-0 32-1 32-2 33-1 33-2
Self-renewing 4	27-0 28-0 29-0 29-2 30-0 30-2 31-0 31-1 31-2 32-0 32-1 32-2 33-1 33-2
Differentiating 1	29-0 30-0 30-2 31-0 31-1 31-2 32-1 32-2 33-1 33-2 34-2
Differentiating 2	29-0 29-2 30-0 30-2 31-0 31-1 31-2 32-1 32-2 33-1 33-2 34-1 34-2
Differentiating 3	29-0 30-0 30-2 31-1 31-2 32-1 32-2 33-1 33-2 34-2
Differentiating 4	28-0 29-0 29-2 30-0 30-2 31-1 31-2 32-1 32-2 33-1 33-2 34-1 34-2

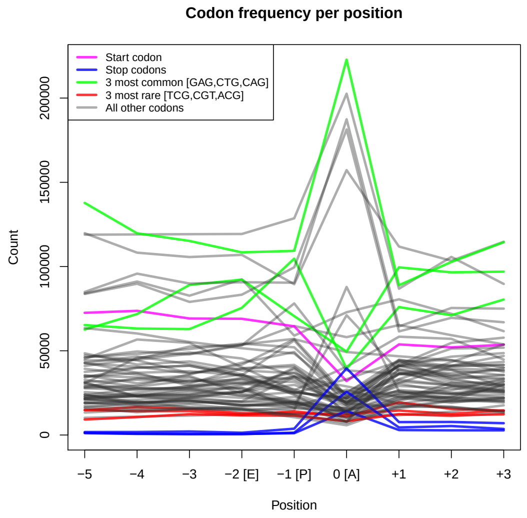

Figure S2: Codon counts across all positions for the *Differentiating 1* sample.

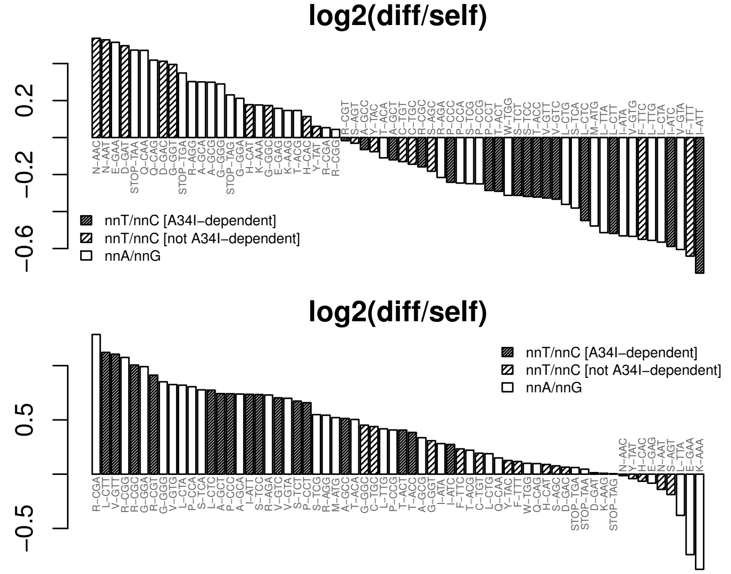

Figure S3: Codon enrichment differences at the ribosome P- (top) and A-site (bottom) between self-renewing (self) and differentiating (diff) cells. Codons that require an inosine-modified tRNA for their translation (dark) and C- and T-ending codons that do not require inosine (dashed) are highlighted. Values shown are log2-transformed.

The four replicates were combined and codon enrichment, e, was calculated for each site, s, using the equation introduced in Bornelöv *et al.* (2019):

$$e_c^s = \log_2\left(\frac{freq_{\mathrm{s}}(c)}{\frac{1}{9}\sum_{i\in\{-5,\ldots,3\}} freq_i(c)}\right) \quad (1)$$

The results generated by CONCUR confirmed the previous finding that codons that require inosine for their translation are depleted at the P-site and enriched at the A-site following exit from pluripotency (Fig. S3). However, compared to the previously published analysis the differences were more pronounced and consistent at the ribosome A-site. The codon-anticodon base pairing at the ribosome A-site was previously suggested to drive this effect (Bornelöv *et al.*, 2019) and displayed a very strong enrichment of A34I-dependent codons in the differentiating state.

This further suggest that considering all read lengths and frames–as done by CONCUR–was more successful than the previoulsy used method to select the dominating frame for the read lengths that showed the strongest periodicity.

1.2 Disruption of tRNA thiolation in yeast

1.2.1 Data and processing

In Eukaryotes, the U_{34} base in the tRNA anticodon loop carries a 5-methoxy-carbonylmethyl (mcm^5) or a 5-carbamoylmethyl (ncm^5) group, modifications that require the Elongator (Elp) complex. The mcm^5 group is further modified by the addition of a 2-thio group (s^2) in $tRNA^{Lys}_{AAA}$, $tRNA^{Gln}_{CAA}$ and $tRNA^{Glu}_{GAA}$ to produce $mcm^5s^2U_{34}$, a modification that is universally conserved (Grosjean *et al.*, 2014). Thiolation requires the combined action of Uba4, Urm1, Ncs2, and Ncs6 and the disruption of thiolation by knockdown of these proteins causes the translating ribosome to stall in *Saccharomyces cerevisiae* and *Caenorhabditis elegans* (Nedialkova and Leidel, 2015). This effect is detectable using ribosome profiling as an enrichment of reads with these codons occupying the ribosome A-site.

To validate that CONCUR could be used to detect those differences, we used the ribosome profiling data for yeast from Nedialkova and Leidel (2015) (data accessible at NCBI GEO database (Edgar *et al.*, 2002), accession GSE67387). The trimmed reads were first aligned to a set of known rRNA and tRNAs (selected from the UCSC RepeatMasker tracks) and this was followed by alignment of all unmapped reads to the the yeast (sc3) reference genome using TopHat2 (v2.1.0) (Kim *et al.*, 2013). No novel junctions were permitted in the alignment to rRNAs and tRNAs. An index with known transcripts based on Ensembl Release 40 annotations were used for the genomeic alignment, novel splice junctions were permitted, and multi-mapping reads were discarded. The resulting BAM files were used as input to CONCUR.

1.2.2 Results

CONCUR was used to select read lengths and frames (Table S2), and to calculate the best shifts. The codon counts derived from the selected reads displayed a strong correlation with each other at both the P- and the A-sites (Fig. S4). An example of the resulting codon counts across all positions is shown in Fig. S5.

Table S2: Selection of reads in yeast

Sample	Selected lengths and frames
ncs2.YPD.rep1	26-0 27-2 28-1 28-2 29-1 30-0 30-1 30-2 31-2
ncs2.YPD.rep2	26-0 27-0 27-2 28-1 29-1 29-2 30-0 30-2 31-0 31-2 32-2
ncs2.YPD.rep3	26-0 26-1 27-0 28-0 28-1 29-1 30-0 30-2 31-0 31-1 31-2 32-1 32-2
WT.YPD.rep1	26-0 27-2 28-1 29-2 30-0 30-2 31-2
WT.YPD.rep2	26-0 27-1 28-0 28-1 28-2 29-0 29-2 30-0 30-1 30-2 31-2
WT.YPD.rep3	25-1 25-2 26-0 26-1 27-0 28-1 29-0 29-1 29-2 30-0 30-1 30-2 31-0 31-1 31-2 32-1 32-2 33-0

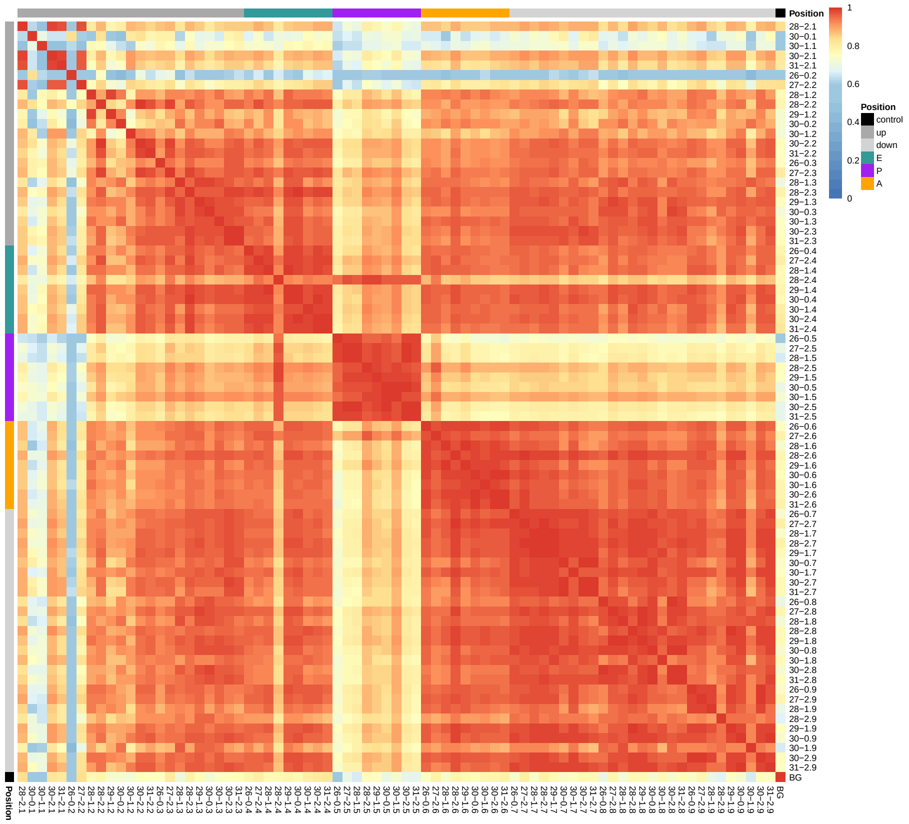

Figure S4: Correlation between selected read sets for the *ncs2.YPD.rep1* sample across all read positions.

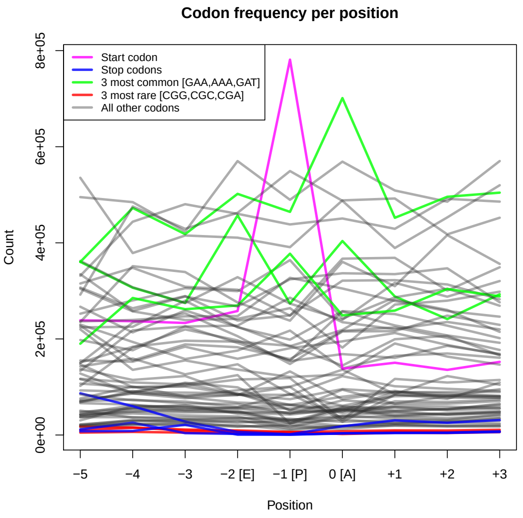

Figure S5: Codon counts across all positions for the *ncs2.YPD.rep1* sample.

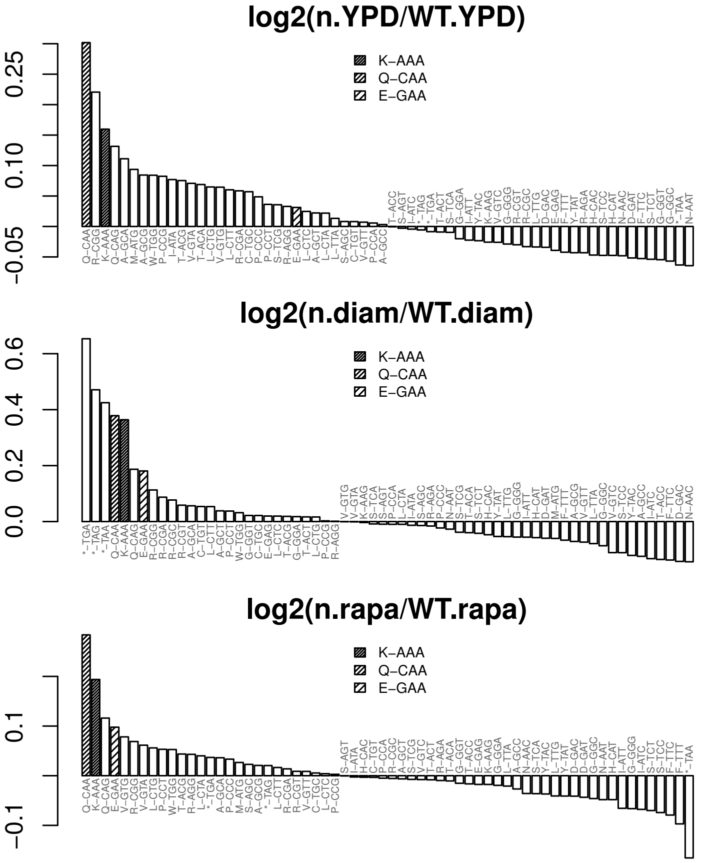

Figure S6: Codon enrichment differences at the ribosome A-site between *ncs2* knockout (n) and wildtype (WT) yeast under normal growth conditions (top), or during stress induced by diamide (middle) or rapamycin (bottom). The three codons that require a modified tRNA are highlighted. Values shown are log2-transformed.

The three replicates were combined and codon enrichment, e, was calculated as described above. The results from CONCUR confirmed that the ribosome stalls at $\text{tRNA}^{\text{Lys}}_{\text{AAA}}$ and $\text{tRNA}^{\text{Gln}}_{\text{CAA}}$ in both normal conditions and during stress induced by diamide or rapamycin (Fig. S6). Furthermore, knockout of elp6 resulted in similar stalling (Fig. S7), which is present both under normal and stress conditions as previously described (Nedialkova and Leidel, 2015).

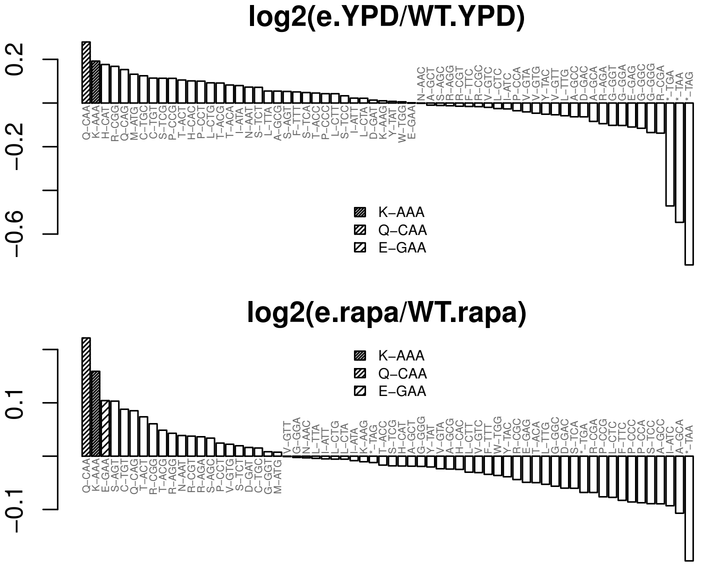

Figure S7: Codon enrichment differences at the ribosome A-site between *elp6* knockout (e) and wildtype (WT) yeast under normal growth conditions (top) or during stress induced by rapamycin (bottom). The three codons that require a modified tRNA are highlighted. Values shown are log2-transformed.

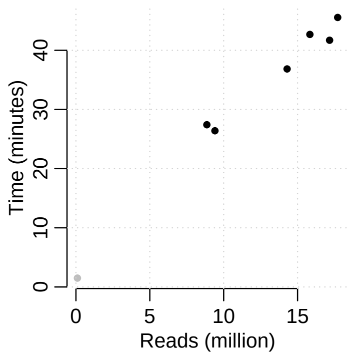

Figure S8: Runtime for six full yeast samples (black) and one yeast sample downsampled to 100,000 reads (grey). Approximately 2.7 minutes were required per million reads processed.

1.2.3 Runtime

Each instance of CONCUR is running as a single-threaded process. The runtime for the yeast samples listed in Table S2 was measured to 26-45 minutes (at 3.6GHz) per sample. The runtime was highly correlated to the number of reads, with approximately 2.7 minutes required per million reads (Fig. S8). A typical ribosome profiling sample with 10 millions uniquely aligned reads would therefore require around 27 minutes (0.45 core-hours) to process.

2 Comparing CONCUR to other methods

2.1 Overview of methods for P-site offset estimation

Identifying P-site offsets is the first step required to estimate codon usage from Ribo-seq data. While there are several Ribo-seq pipelines available dedicated for tasks such as quality control or ORF detection, most of them work as "black boxes" with respect to any P-site offset calculation. Some pipelines rely on external tools for offset calculation, typically Plastid or Ribowaltz (Dunn and Weissman, 2016; Lauria *et al.*, 2018), or have implemented heuristics similar to Plastid, such as identifying the 5' end read count maxima. Other approaches use a pre-defined position for the P-site, such as RiboVIEW (Legrand and Tuorto, 2020) that uses a fixed -12 offset and SPECtre (Chun *et al.*, 2016) that uses the midpoint of the read.

Table S3 provides an overview of existing tools developed for P-site offset calculations as well as general Ribo-seq pipelines that have implemented their own offset calculation. Most tools, including CONCUR, require sequence-independent cleavage of the ribosome-protected fragments with an RNase. RiboProP (Zhao *et al.*, 2019) is the only tool listed that is aimed towards MNase digested Ribo-seq and would be the method of choice for such data.

Table S3: Dedicated tools and general Ribo-seq pipelines that can perform P-site offset estimation.

**Name**	**Frame**[a]	**Codon usage**	**Alignment**	**Species**	**Documented**[b]	**Reference**
CONCUR	All	Yes	Genome	Any	Yes	*this study*
ORFik	Single	Yes	Genome	Any	Yes	github.com/Roleren/ORFik
Plastid	Single	No	Both	Any	Yes	Dunn and Weissman (2016)
Ribodeblur	All	A-site only	Transcriptome	Yeast	Yes	Wang *et al.* (2017)
RiboProfiling	Single	Yes	Genome	Multiple	Yes	Popa *et al.* (2016)
RiboProP	Single	No	Both	Bacteria	Yes	Zhao *et al.* (2019)
RiboStreamR	Single	No	Genome	Any	No	Perkins *et al.* (2019)
Ribo-TISH	Single	No	Genome	Any	Yes	Zhang *et al.* (2017)
riboWaltz	Single	P-site only	Transcriptome	Any	Yes	Lauria *et al.* (2018)
RUST	Single	No	Genome	Any	Partially	O'Connor *et al.* (2016)
Shoelaces	Single	No	Genome	Any	Yes	Birkeland *et al.* (2018)
SPECtre	Single	No	Genome	Any	No	Chun *et al.* (2016)

[a]Description of whether the tools handles multiple reading frames per read length.
[b]This refer only to whether the P-site offset estimation is described in a publication, tool documentation or source code.

Section 2.2 provides an overview of some key differences between CONCUR and existing tools. Furthermore, in Section 2.3, we compared CONCUR to Plastid (Dunn and Weissman, 2016), Shoelaces (Birkeland *et al.*, 2018) and ORFik [`https://github.com/Roleren/ORFik`] for P-site offset calculation and evaluated the results by the consistency in the estimated codon usage.

2.2 Key features and differences to other tools

2.2.1 Simple processing and input format

While there are some existing tools dedicated to P-site offset calculations (Dunn and Weissman, 2016; Lauria *et al.*, 2018; Wang *et al.*, 2017; Birkeland *et al.*, 2018), most other tools perform P-site offset calculations as part of a pipeline and are often not able to run offset calculations separately. Like other dedicated tools, CONCUR relies solely on a BAM file with reads aligned to the genome, which makes it fast, easy to run and suitable to both run independently or to be included in a pipeline.

CONCUR is currenly based on alignments to a reference genome, but support for transcriptome alignment can be added by running concur_install_genome.pl with a custom GTF file representing the coding sequences in the transcriptome.

2.2.2 Focus on codon usage estimates

CONCUR was built with the primary focus not only to offset and filter reads correctly, but more importantly, to calculate and return codon usage at the E-, P-, and A-site as well as at the flanking positions. Existing tools that are able to calculate offsets do not automatically remove reads that are not in frame with those offsets, makes codon usage measurements impossible without additional work. Furthermore, the stringent filtering in CONCUR makes the final read sets more consistent with each other and suitable for codon usage calculations.

There is a need for Ribo-seq tools aimed at specific tasks in a well-described and rigorous manner. CONCUR is filling this gap by providing a methodology aimed specifically at codon usage calculations.

2.2.3 Correct inclusion of multiple frames per read length

A notable difference between CONCUR and other tools performing P-site offset calculation is that CONCUR does not assume all reads of the same length to have the same offset. The first step of CONCUR is to identify reads that intersect with coding sequences to determine the reading frame of their 5' ends. Reads of the same length–but different frames–are identified and treated as separate read sets in all subsequent analyses.

To illustrate this point, we can consider the yeast study in Section 1.2. CONCUR determined reads of 30 nt in length in the ncs2.YPD.rep1 replicate to have an offset of -12, -14, and -13 for frame 0, 1, and 2, respectively. All three frames gave similar codon usage signals as shown in the pairwise correlation plot (Fig. S4). Notably, the most common frame for length 30 was frame 2, with only 50% of the reads. In other words, half of the reads would either have been processed using an incorrect offset, or been excluded, in case a single frame had been used. All tools shown in Table S3 except CONCUR and Ribodeblur assumes a single frame.

2.3 Benchmarking of P-site offset estimation

2.3.1 Running CONCUR, Plastid, ORFik, and Shoelaces

We compared the P-site offsets estimated by CONCUR, to those estimated by Plastid, ORFik, and Shoelaces. We selected these three tools as they all have a well documented offset estimation procedure, they are compatible with genomic alignments, they work for all species, and they did not require us to run a whole pipeline to calculate the P-site offsets. We did not find any other tool fulfilling all these criteria.

Plastid (v0.4.7) was run with "--min_length 22", "--max_length 36" and "--default 100" to allow for non-conclusive read lengths to be identified and omitted from the comparison. CONCUR (v0.9), ORFik (v1.6.9) and Shoelaces (v1.6) were run with default read length settings and any read length that passed filters were included in the results. None of the tools returned any offsets for reads of length <25 or >36, with the exception of a single sample that had an offset estimated for length 38 by ORFik. We therefore focused on P-site offsets for reads of length 25-36.

Plastid, ORFik and Shoelaces report up to one offset per read length, but do not report any reading frame information for that offset. When comparing these tools to CONCUR, we calculated the frame manually as offset *modulo* 3. Only reads with the expected frame were included in the codon usage estimation that was used to validate the offsets. Excluding reads from other frames is neccessary for any codon usage analysis, and omitting this step would have strongly reduced the performance compared with CONCUR.

2.3.2 Benchmarking results

Table S4 and Table S5 show all P-site offsets reported by CONCUR, Plastid, ORFik, and Shoelaces, respectively, calculated for six yeast samples previously covered in Section 1.2. To validate the offsets we compared the resulting P-site codon usage across different read lengths (and frames, when applicable) to each other. Read sets with P-site codon usage highly correlated to P-site codon usage from other reads sets were considered to be *consistent*. Read sets with very different P-site codon usage profiles were considered *inconsistent*, whereas those with only partially different profiles were considered *partially consistent*. The number of consistent, partially consistent and inconsistent read sets for each tool is summarized in Table S6 (top).

CONCUR performed slightly better than Plastid and Shoelaces, reporting on average 10.3 consistent reads sets, compared with 8.7 for both Plastid and Shoelaces. ORFik performed worse with only 3.8 consistent read sets reported per sample. Moreover, CONCUR selected the fewest partially consistent or inconsistent read sets, presumably due to its stringent filtering and validation steps. It should be noted that the samples analyzed here had a low 3nt periodicity. This may explain why ORFik, which uses a Fourier transform, performed less well than the other tools.

The number of reads per read set varies in Ribo-seq data, making it more important to estimate offsets correctly for more abundant read sets than for less abundant ones. To evaluate how many reads would have a correct offset for codon usage analysis, we compared the number of reads that were selected from the consistent, partially consistent, and inconsistent read sets, respectively (Table S6, bottom). CONCUR retained more reads from consistent read sets than any other tool (9.2 million reads, compared with 6.8 million reads for Plastid and 6.7 million reads for Shoelaces). This difference is likely due to the use of multiple frames by CONCUR, thereby avoiding the uneccessary exclusion of read sets in alternative frames of abundant read lengths.

Together, the examples illustrated how using multiple reading frames combined with stringent filtering makes CONCUR well-suited for codon usage analyses.

Table S4: P-site offset estimation by CONCUR, Plastid, ORFik, and Shoelaces in three *ncs2* knockout samples. Offsets that gave consistent counts at the P-site are shown in black, partially consistent ones in orange, and inconsistent ones in red. Additionally, initial offsets that were later filtered and not included in the final report from CONCUR are shown in light grey (excluded during initial read shift estimation) or grey (excluded during the validation) and italic font.

	**CONCUR**			**Plastid**			**ORFik**			**Shoelaces**		
"ncs2.YPD.rep1"	**Frame**			**Frame**			**Frame**			**Frame**		
**Length**	**0**	**1**	**2**	**0**	**1**	**2**	**0**	**1**	**2**	**0**	**1**	**2**
25		*-8*	*-7*		-8						-8	
26	-9	*-8*	*-10*		-8				-13	-9		
27	*-9*	*-11*	-10	-9					-13	-9		
28	*-12*	-11	-13	-12					-13	-12		
29	*-12*	-11	*-13*			-13			-13			-13
30	-12	-14	-13			-13		-14				-13
31	*-12*	*-14*	-13			-13		-14				-13
32	*-15*	*-14*	*-13*		-14		-15				-14	
33	*-15*	*-14*			-14				-16		-14	
34						-16			-16			-10
35						-16			-1			
36												

	**CONCUR**			**Plastid**			**ORFik**			**Shoelaces**		
"ncs2.YPD.rep2"	**Frame**			**Frame**			**Frame**			**Frame**		
**Length**	**0**	**1**	**2**	**0**	**1**	**2**	**0**	**1**	**2**	**0**	**1**	**2**
25	*-6*		*-7*			-7						-7
26	-9	*-8*	*-7*		-8						-8	
27	-9	*-8*	-10	-9				-14		-9		
28	*-12*	-11	*-10*	-12					-13	-12		
29	*-12*	-11	-13	-12					-13			-13
30	-12	*-14*	-13			-13		-14				-13
31	-12	*-14*	-13			-13		-14				-13
32	*-15*	*-14*	-13			-13	-15					-13
33	*-15*	*-14*	*-13*		-14		-15				-14	
34				-15				-16		-15		
35						-16				-12		
36									-5			

	**CONCUR**			**Plastid**			**ORFik**			**Shoelaces**		
"ncs2.YPD.rep3"	**Frame**			**Frame**			**Frame**			**Frame**		
**Length**	**0**	**1**	**2**	**0**	**1**	**2**	**0**	**1**	**2**	**0**	**1**	**2**
25	*-6*		*-7*			-7	-9					-7
26	*-9*	-8	*-7*		-8							
27	-9	*-8*	*-10*	-9				-14		-9		
28	-9	-11	*-10*	-9				-14		-9		
29	*-12*	-11	*-13*	-12				-14		-12		
30	-12	*-14*	-13			-13		-14		-12		
31	-12	*-14*	-13			-13		-14				-13
32	*-12*	*-14*	-13			-13	-15					-13
33	*-15*	*-14*	*-13*		-14		-15				-14	
34	*-15*	*-14*	*-16*	-15					-16	-15		
35						-10						
36									-7			-11

Table S5: P-site offset estimation by CONCUR, Plastid, ORFik, and Shoelaces in three wildtype samples. Offsets that gave consistent counts at the P-site are shown in black, partially consistent ones in orange, and inconsistent ones in red. Additionally, initial offsets that were later filtered and not included in the final report from CONCUR are shown in light grey (excluded during initial read shift estimation) or grey (excluded during the validation) and italic font.

	**CONCUR**			**Plastid**			**ORFik**			**Shoelaces**		
”WT.YPD.rep1”	**Frame**			**Frame**			**Frame**			**Frame**		
**Length**	**0**	**1**	**2**	**0**	**1**	**2**	**0**	**1**	**2**	**0**	**1**	**2**
25					-8							
26	-9	*-8*	*-10*		-8				-13		-8	
27	*-9*	*-8*	-10	-9					-13	-9		
28	*-12*	-11	*-13*	-12					-13	-12		
29	*-12*	*-11*	-13	-12					-13	-12		
30	-12	*-14*	-13			-13		-14				-13
31	*-12*	*-14*	-13			-13		-14				-13
32	*-15*	*-14*	*-13*		-14		-15				-14	
33	*-15*	*-14*			-14				-16		-14	
34						-10						-10
35												
36												
	**CONCUR**			**Plastid**			**ORFik**			**Shoelaces**		
”WT.YPD.rep2”	**Frame**			**Frame**			**Frame**			**Frame**		
**Length**	**0**	**1**	**2**	**0**	**1**	**2**	**0**	**1**	**2**	**0**	**1**	**2**
25						-7					-8	
26	-9	*-8*	*-10*		-8				-13	-9		
27	*-9*	-11	*-10*			-10			-13	-9		
28	-12	-11	-13	-12					-13	-12		
29	-12	*-11*	-13	-12					-13			-13
30	-12	-14	-13			-13		-14				-13
31	*-12*	*-14*	-13			-13		-14				-13
32	*-15*	*-14*	*-13*		-14		-15				-14	
33	*-15*	*-14*			-14				-16		-14	
34						-10			-16			-10
35												-10
36												
	**CONCUR**			**Plastid**			**ORFik**			**Shoelaces**		
”WT.YPD.rep3”	**Frame**			**Frame**			**Frame**			**Frame**		
**Length**	**0**	**1**	**2**	**0**	**1**	**2**	**0**	**1**	**2**	**0**	**1**	**2**
25	*-6*	-8	-7			-7						-7
26	-9	-8	*-7*		-8						-8	
27	-9	*-8*	*-10*	-9					-13	-9		
28	*-9*	-11	*-10*			-10		-14		-9		
29	-12	-11	-13	-12				-14		-12		
30	-12	*-14*	-13			-13		-14		-12		
31	-12	-14	-13			-13		-14				-13
32	*-12*	-14	-13			-13	-15					-13
33	-15	*-14*	*-13*		-14		-15				-14	
34				-15					-16	-15		
35						-10						
36												

Table S6: Summary of the benchmarking results. All numbers shown are mean values across the six Ribo-seq libraries.

	**CONCUR**	**Plastid**	**ORFik**	**Shoelaces**
Consistent read sets	10.3	8.7	3.8	8.7
Partially consistent read sets	0.5	1.0	1.2	0.7
Inconsistent read sets	0.0	0.8	4.0	0.8
	**CONCUR**	**Plastid**	**ORFik**	**Shoelaces**
Reads overlapping CDS	14,508,258	14,508,258	14,508,258	14,508,258
Consistent reads selected	9,177,421	6,773,610	2,420,551	6,682,485
Partially consistent reads selected	28,834	65,701	51,475	72,290
Inconsistent reads selected	0	558	226,595	410

References

Birkeland,Å., Chyżyńska,K., Valen,E. (2018) Shoelaces: an interactive tool for ribosome profiling processing and visualization. *BMC Genomics*, **19**:543, doi:10.1186/s12864-018-4912-6.

Bornelöv,S., Selmi,T., Flad,S., *et al.* (2019) Codon usage optimization in pluripotent embryonic stem cells. *Genome Biology*, **20**:119, doi:10.1186/s13059-019-1726-z.

Chun,S.Y., Rodriguez,C.M., Todd,P.K. *et al.* (2016) SPECtre: a spectral coherence-based classifier of actively translated transcripts from ribosome profiling sequence data. *BMC Bioinformatics*, **17**:482, doi:10.1186/s12859-016-1355-4.

Dunn,J.G., Weissman,J.S. (2016) Plastid: nucleotide-resolution analysis of next-generation sequencing and genomics data. *BMC Genomics*, **17**:958, doi:10.1186/s12864-016-3278-x.

Edgar,R., Domrachev,M., Lash,AE. (2002) Gene Expression Omnibus: NCBI gene expression and hybridization array data repository. *Nucleic Acids Res*, **30**(1):207-10, doi:10.1093/nar/30.1.207.

Grosjean,H., Breton,M., Sirand-Pugnet,P., *et al.* (2014) Predicting the Minimal Translation Apparatus: Lessons from the Reductive Evolution of Mollicutes. *PLoS Genet* **10**(5):e1004363, doi:10.1371/journal.pgen.1004363.

Kim,D., Pertea,G., Trapnell,C., *et al.* (2013) TopHat2: accurate alignment of transcriptomes in the presence of insertions, deletions and gene fusions. *Genome Biology*, **14**:R36, doi:10.1186/gb-2013-14-4-r36.

Lauria,F., Tebaldi,T., Bernabò,P., *et al.* (2018) riboWaltz: Optimization of ribosome P-site positioning in ribosome profiling data. *PLoS Comput Biol*, **14**(8):e1006169, doi:10.1371/journal.pcbi.1006169.

Legrand,C., Tuorto,F. (2020) RiboVIEW: a computational framework for visualization, quality control and statistical analysis of ribosome profiling data. *Nucleic Acids Res*, **48**(2):e7, doi:10.1093/nar/gkz1074.

Nedialkova,C., Leidel,S.A. (2015) Optimization of Codon Translation Rates via tRNA Modification Maintains Proteome Integrity. *Cell*, **161**:1606-1618, doi:10.1016/j.cell.2015.05.022.

O'Connor,P.B.F., Andreev,D. and Baranov,P.V. (2016) Comparative survey of the relative impact of mRNA features on local ribosome profiling read density. *Nature Comm*, **7**:12915, doi:10.1038/ncomms12915.

Perkins,P., Mazzoni-Putman,S., Stepanova,A., *et al.* (2019) RiboStreamR: a web application for quality control, analysis, and visualization of Ribo-seq data. *BMC Genomics*, **20**:422, doi:10.1186/s12864-019-5700-7.

Popa,A., Lebrigand,K., Paquet,A. *et al.* (2016) RiboProfiling: a Bioconductor package for standard Ribo-seq pipeline processing [version 1; peer review: 3 approved]. *F1000Research*, **5**:1309, doi:10.12688/f1000research.8964.1.

Wang,H., McManus,J., Kingsford,C. (2017) Accurate Recovery of Ribosome Positions Reveals Slow Translation of Wobble-Pairing Codons in Yeast. *J Comput Biol*, **24**:6, doi:10.1089/cmb.2016.0147.

Zhang,P., He,D., Xu,Y. *et al.* (2017) Genome-wide identification and differential analysis of translational initiation. *Nat Commun*, **8**:1749, doi:10.1038/s41467-017-01981-8.

Zhao,D., Baez,W.D., Fredrick,K., Bundschuh,R. (2019) RiboProP: a probabilistic ribosome positioning algorithm for ribosome profiling. *Bioinformatics*, **35**:9, 1486-1493, doi:10.1093/bioinformatics/bty854.
